# Adverse effects in hematologic malignancies treated with chimeric antigen receptor (CAR) T cell therapy: a systematic review and Meta-analysis

**DOI:** 10.1186/s12885-021-09102-x

**Published:** 2022-01-24

**Authors:** Wenjing Luo, Chenggong Li, Yinqiang Zhang, Mengyi Du, Haiming Kou, Cong Lu, Heng Mei, Yu Hu

**Affiliations:** 1 Institute of Hematology, Union Hospital, Tongji Medical College, Huazhong University of Science and Technology, 430022 Wuhan, China; 2 Hubei Clinical Medical Center of Cell Therapy for Neoplastic Disease, 430022 Wuhan, China

**Keywords:** Chimeric antigen receptor, Hematological malignancies, Hematologic toxicity, Meta-analysis, Review

## Abstract

**Background:**

Recently, chimeric antigen receptor-modified (CAR) T cell therapy for hematological malignancies has shown clinical efficacy. Hundreds of clinical trials have been registered and lots of studies have shown hematologic toxic effects were very common. The main purpose of this review is to systematically analyze hematologic toxicity in hematologic malignancies treated with CAR-T cell therapy.

**Methods:**

We searched databases including PubMed, Web of Science, Embase and Cochrane up to January 2021. For safety analysis of overall hematologic toxicity, the rate of neutrophil, thrombocytopenia and anemia were calculated. Subgroup analysis was performed for age, pathological type, target antigen, co-stimulatory molecule, history of hematopoietic stem cell transplantation (HSCT) and prior therapy lines. The incidence rate of aspartate transferase (AST) increased, alanine transaminase (ALT) increased, serum creatine increased, APTT prolonged and fibrinogen decreased were also calculated.

**Results:**

Overall, 52 studies involving 2004 patients were included in this meta-analysis. The incidence of any grade neutropenia, thrombocytopenia and anemia was 80% (95% CI: 68–89%), 61% (95% CI: 49–73%), and 68% (95%CI: 54–80%) respectively. The incidences of grade ≥ 3 neutropenia, thrombocytopenia and anemia were 60% (95% CI: 49–70%), 33% (95% CI: 27–40%), and 32% (95%CI: 25–40%) respectively. According to subgroup analysis and the corresponding Z test, hematological toxicity was more frequent in younger patients, in patients with ≥4 median lines of prior therapy and in anti-CD19 cases. The subgroup analysis of CD19 CAR-T cell constructs showed that 41BB resulted in less hematological toxicity than CD28.

**Conclusion:**

CAR-T cell therapy has dramatical efficacy in hematological malignancies, but the relevant adverse effects remain its obstacle. The most common ≥3 grade side effect is hematological toxicity, and some cases die from infections or severe hemorrhage in early period. In long-term follow-up, hematological toxicity is less life-threatening generally and most suffered patients recover to adequate levels after 3 months. To prevent life-threatening infections or bleeding events, clinicians should pay attention to intervention of hematological toxicity in the early process of CAR-T cell therapy.

**Supplementary Information:**

The online version contains supplementary material available at 10.1186/s12885-021-09102-x.

## Background

Hematological malignancies accounted for 1.2 million, that was around 7%, newly diagnosed cancer cases every year worldwide [1]. Among them, lymphocytic leukemia, lymphoma and multiple myeloma (MM) represent a large part. Chemotherapy, as a traditional and common treatment for them, is being replaced gradually by some novel therapies, like chimeric antigen receptor-modified (CAR-T) cell therapy.

CAR-T cells are produced strictly ex-vivo and then infused to patients by intravenous injection. The CARs, recognizing their targets by a specific mechanism distinct from classic TCRs, are comprised of an antigen-specific single-chain variable fragment (scFv) that is fused to an internal T-cell signaling domain and costimulatory molecules like CD28 or 41BB [2]. The development of CAR-T cell therapy was a wave of optimism for selected hematological malignancies in the past decades. Meanwhile, cytokine release triggered by CAR-T cell activation, expansion and cytotoxicity, leads to CRS, immune effector cell-associated neurotoxicity syndrome (ICANS) and even hematological toxicities [3, 4]. Adverse effects related to CAR-T cell therapy should be paid attention to, and there are already some reviews reporting the overall rate of CRS and ICANS. And hematological toxicity is the most common grade ≥ 3 AE in CAR-T cell therapy [5]. Given that hepatotoxicity, nephrotoxicity and coagulation disorders are not rare in the treatment of hematological malignancies, we analyzed these incidences as the secondary outcome. The analysis of the landscape of hematological toxic effects associated with CAR-T cell therapy seems to be extremely significant.

We searched databases including PubMed and Web of Science to explore the adverse effects during the CAR-T cell therapy, and 52 studies involving 2004 patients were included in this meta-analysis. We mainly analyzed hematological toxicity, and we also conducted subgroup analysis. We aimed to provide some references for CAR-T cell therapy and draw clinicians’ attention to AEs associated with CAR-T cell therapy, besides CRS and neurotoxicity.

## Materials and methods

This study is registered in International Prospective Register of Systematic Reviews (PROSPERO) and the number is CRD 42021237114. We did our meta-analysis and systematic review in accordance with the PRISMA (Preferred Reporting Items for Systematic Reviews and Meta-Analyses) guidelines [6] and the checklist is shown in Supplementary Material.

### Search strategy

We searched PubMed, Web of Science, Embase and Cochrane up to January 2021, and the terms for the literature search were “chimeric antigen receptor”, “CAR-T”, “chimeric antigen receptor-modified T cell therapy”, “blood system toxicity”, “hematopoietic system toxicity”, “hematological toxicity”, “adverse effects”, “side effects”, “leukemia”, “multiple myeloma”, “lymphoma” and “hematological malignancies”. To guarantee comprehensive search and to include all potentially relevant studies, we examined related meta-analysis and cross-referenced the references of identified articles. The search results were imported in Endnote X9 and duplicates were identified and removed through Endnote X9 and manually. Two independent researchers (Luo WJ and Mei H) screened retrieved documents and assessed independently full texts of articles on the basis of prespecified inclusion criteria. All disagreements were resolved by discussion with the third researcher (Hu Y).

### Selection criteria

#### Inclusion criteria

We included both articles published in journal and abstracts from conference proceedings, which reported the incidence rate of hematological toxicity in patients with CAR-T cell therapy. Both single-arm trials and retrospective studies were included. Case-series with detailed information of treatment and outcome were also included. We analyzed the most recently updated results of each included clinical trial, whether reported in published articles or conference proceedings.

#### Exclusion criteria

We excluded studies published in languages other than English and Chinese, and those focusing on the efficacy or safety of combinations of CAR-T cell therapy and other therapies. Studies with insufficient data where our aimed AEs were not reported, irrelevant studies, and studies with two or fewer patients were excluded. Studies with the same NCT number were screened, and we excluded these reports with the shorter follow-up. Meanwhile, clinical guidelines, consensus documents and systematic reviews were excluded from our meta-analysis.

### Data extraction

Two investigators independently reviewed and extracted the following information: study characteristics (first author, publication year, the number of included patients, ClinicalTrials.gov number, research design and the selected AEs criteria), patients characteristics (gender, age, pathological type, previous HSCT and prior therapy lines), intervention (pre-infusion conditioning, CAR-T cell dose, target selection and costimulatory molecule), the incidence rate (neutropenia, thrombocytopenia, anemia, AST increasement, ALT increasement, serum increasement, APTT prolongation and fibrinogenopenia), and the onset and recovery time of hematological toxicity. And we two stored the information using Microsoft Excel for analysis. Disagreements were settled by discussion with the third reviewer.

### Methodological quality of the included studies

We used a specific tool for evaluating the methodological quality of the non-comparative studies [7]. This tool is categorized into four domains: selection of patients, ascertainment of exposure and outcome, causality and reporting [7]. We assessed methodological quality of each study by grading the risk of bias as low (score of 0–1), moderate (score of 2–3) and high (score of 4).

### Statistical analysis

We used the “Meta” and “Metafor” packages in the R-4.0.3 statistical software to analyze therapeutic safety. The incidence rates and relevant 95% confidence intervals (CIs) were calculated to estimate pooled results from studies. In case of no obvious heterogeneity (I^2^ < 50% and *P* > 0.05 in the Q test), the results from fixed-effects model were reported in our meta-analysis. Otherwise, the results from random-effects model were reported. All pooled results with *P*-values ≤0.05 were considered statistically significant. We performed the Egger’s test to assess statistically the publication bias (*P* > 0.05 was considered indicative of no significant publication bias), and funnel plots were constructed for providing a visual analysis of publication bias. Sensitivity analysis was conducted for estimating the effect on the overall rates of neutropenia, thrombocytopenia and anemia, with removal of the corresponding studies one by one. Subgroup analysis by age (< 45 vs. ≥45 and < 60 vs. ≥60), target antigen selected (CD19 vs. no CD19), co-stimulatory molecule (41BB vs. CD28), proportion of previous HSCT (< 50% vs. ≥50%), and the median lines of prior therapy (< 4 vs. ≥4) was performed to explore the sources of heterogeneity, and Z test was conducted for comparing the merged incidence rates between subgroups.

## Results

### Literature search and study characteristics

**Fig. 1:**
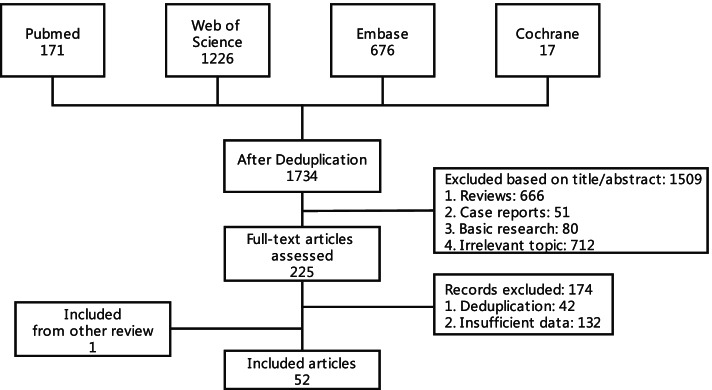
Flowchart describing the literature selection process

Two thousand ninety potentially relevant studies were retrieved, and 356 studies were de-duplicated by EndNote X9. By screening titles and abstracts, 666 reviews, 51 case reports, 80 basic studies and 712 studies with irrelevant topic were excluded. After full texts were carefully reviewed, among studies based on the same data sources, we only included one with the most recent updated results of clinical trials. Besides, 132 studies with insufficient data were excluded. One additional study was included by cross searching the references of previous meta-analysis. Finally, 52 eligible studies involving 2004 patients were included [8–59]. The flowchart describing the literature selection process is presented in Fig. 1. The characteristics of the included studies is shown in Table 1. Of the included studies, 47 (90%) explored the incidence rate of hematological toxicity, 20 (38%) explored the hepatic toxicity, 10 (19%) explored the renal toxicity and 11 (21%) explored the coagulation dysfunction related to CAR-T cell therapy. The detailed features of the included patients in their corresponding studies are presented in Table 2**.** As shown, the target patients of included studies were those with lymphoma, leukemia or MM. The proportion of male was 39–100%; the median patients age ranged from 7.5 to 67 years; the median lines of prior therapy ranged from 3 to 7; and the proportion of prior HSCT was 0–100%. Based on the assessment of quality, the included studies had a risk bias of low or moderate **(**Table 3**)**.

**Table 1:** Basic characteristics of the included studies

Name	Type of literature	Journal	Year Published	Trial sequence	Design	Sample	Pre-infusion conditioning	Dose	Target	Costimulatory domain	AEs criteria
Ying Zhita; ^a^	Journal	Molecular Therapy-Oncolytics	2019	NCT03528421	phase 1/2	3	CF	5*10^5/kg	CD19	CD28	CTCAE v5.0
Ying Zhita;^a^	Journal	Molecular Therapy-Oncolytics	2019	NCT03528421	phase 1/2	3	CF	5*10^5/kg	CD19	41BB	Not found
Yan, Zi-Xun	Journal	Clinical Cancer Research	2019	NCT03355859	phase 1	10	CF	(2.5 or 5 or 10) *10^7	CD19	41BB	CTCTAE v4.03
Sang, W	Journal	Cancer Med	2020	NCT03207178	phase 2	21	CF/ifosfamide	CD19: 1.0 (0.2–4.0) *10^6/kg CD20:1.0*(0.1–4.0) *10^6/kg	CD19 + CD22	CD28 + 41BB	CTCTAE v4.03
Tong, C	Journal	Blood	2020	NCT03097770	phase 1/2a	28	CF-based	0.5*10^6–6*10^6/kg	CD19 + CD20	41BB	CTCAE v4.0
Xu, J	Journal	PNAS	2019	NCT03090659	phase 1	17	CF/Cy-based	0.7(0.21–1.52) *10^6/kg	LCAR-B38M	41BB	CTCTAE v4.03
Zhao, W. H	Journal	J Hematol Oncol	2018	NCT03090659	phase1	57	Cy	0.5(0.07–2.1)*10^6/kg	LCAR-B38M	41BB	CTCTAE v4.03
Shah, N. N	Journal	Nature Medicine	2020	NCT03019055	phase 1	22	CF	(2.5 or 7.5 or 25) * 10^5/kg	CD19 + CD20	41BB	CTCAE v5.0
Wang, Y	Journal	Int J Lab Hematol	2020	NCT02782351	phase 1/2	21	CF	1*10^6/kg	CD19	41BB	CTCTAE v4.03
Fried, S.	Journal	Bone Marrow Transplant	2019	NCT02772198	phase1b/2	35	CF		CD19	CD28	Not found
An, F	Journal	Nature Communications	2020	NCT02735291	phase 2	47	CF/VDCP/ Cy	(1–5)*10^6/kg; ≤2*10^9	CD19	41BB	CTCTAE v4.03
Ramos, C. A	Journal	Journal of Clinical Oncology	2020	NCT02690545 NCT02917083	phase 1/2	42	CF/Benda/Benda-Flu	2*10^7cells/m2; 1*10^8cells/m2; 2*10^8 cells/m2	CD30	CD28	CTCAE v4.0
Raje, N	Journal	N Engl J Med	2019	NCT02658929	phase 1	33	CF	50、150、450、800*10^6	BCMA	41BB	CTCTAE v4.03
Abramson, J. S	Journal	lancet	2020	NCT02631044	phase 1	269	CF	(50 or 10 or 150) *10^6	CD19	41BB	CTCTAE v4.03
Wang, M	Journal	N Engl J Med	2020	NCT02601313	phase 2	68	CF	2*10^6/kg	CD19	41BB	CTCTAE v4.03
Cohen, A. D	Journal	J Clin Invest	2019	NCT02546167	phase 1	25	Cy	(1–5)*10^8	BCMA	41BB	CTCAE v4.0
Goto, H	Journal	Int J Clin Oncol	2020	NCT02445248	phase 2	9	CF or Benda	2*(1–4.9)*10^8	CD19	41BB	CTCAE v4.03
Schuster, S. J	Journal	N EngL J Med	2018	NCT02445248	phase 2a	111	CF/Benda	3(0.1–6)*10^8 cells	CD19	41BB	CTCTAE v4.03
Ghorashian, S	Journal	Nat Med	2019	NCT02443831	phase1	14	CF/Cy	10^6/kg or 0.73–0.78*10^6/kg	CD19	41BB	CTCAE v4.03
Maude, S. L	Journal	N Engl J Med	2018	NCT02435849	phase 1/2a	75	CF mainly	2.9(SD1.2)*10^7/kg	CD19	41BB	CTCAE v4.03
Strati, Paolo	Journal	Haematologica	2020	NCT02348216 NCT03153462	ZUMA-1 + ZUMA-9	31	CF	2*10^6/kg	CD19	CD28	CTCTAE v4.03
Locke, F. L	Journal	Lancet Oncol	2019	NCT02348216	phase 1/2	108	CF	2*10^6/kg	CD19	CD28	CTCTAE v4.03
Fry, T. J	Journal	Nature medicine	2017	NCT02315612	phase 1	21		(3 or 10 or 30)*10^5/kg	CD22	41BB	Not found
Ali, S. A	Journal	Blood	2016	NCT02215967	phase 1	12	CF	(0.3 or 1 or 3 or 9)*10^6/kg	BCMA	CD28	CTCAE v4.02
Enblad, Gunilla	Journal	Clin Cancer Res	2018	NCT02132624	phase 1/2a	15	CF	(2–20)*10^7 cells/m2	CD19	CD28 + 41BB	Not found
Schuster, S. J	Journal	N Engl J Med	2017	NCT02030834	case-series	28	Cy/EPOCH/Benda/Radio+Cy/etoposide+Cy/CBP + GEM	5.79(3.08–8.87)*10^6 /Kg	CD19	41BB	Not found
Gardner, R. A	Journal	Blood	2017	NCT02028455	phase 1/2	43	CF/Cy	(1 or 5 or 10)*10^6/kg	CD19	41BB	CTCAE v4
Curran, K. J	Journal	Blood	2019	NCT01860937	phase 1	25	CF/Cy	(1 or 3)*10^6/kg	CD19	CD28	CTCTAE v4.03
Ramos, Carlos A	Journal	Molecular Therapy	2018	NCT01853631	phase 1	16	CF	(1 or 5 or 20)*10^6 cells/m2	CD19	CD28 + 41BB(2nd + 3st generation)	CTCTAE v4
Zhang, W. Y	Journal	Signal Transduct Target Ther	2016	NCT01735604	phase 2a	11	Cy-based	(0.41–1.46)*10^7/ kg	CD20	41BB	CTCAE v3.0
Lee, D. W^b^	Journal	Lancet	2014	NCT01593696	phase 1	19	CF	(1 or 3)*10^6/kg	CD19	CD28	CTCAE v4.02
Geyer, M. B.	Journal	Mol Ther	2018	NCT01416974	phase1	8	Cy	(3 or 10 or 30)*10^7	CD19	CD28	CTCAE v4
Geyer, M. B	Journal	JCI Insight	2019	NCT00466531	phase1	20	Cy,or CF or Benda	(0.4–3.0)*10^7/kg	CD19	CD28	CTCAE v3.0
Sesques, P^c^	Journal	American Journal of Hematology	2020	commercial CAR T cells	retrospectively	33	CF;/Benda	Not found	CD19	41BB	CTCAE v5.0
Sesques, P^c^	Journal	American Journal of Hematology	2020	commercial CAR T cells	retrospectively	28	CF	Not found	CD19	CD28	CTCAE v5.0
Wang, N^d^	Journal	Blood	2019	ChiCTR-OPN-16008526	a pilot study	51	CF	CD19:2.6 ± 1.5*10^6/kg; CD22:2.7 ± 1.2*10^6/kg;	CD19 + CD22	CD28 + 41BB	CTCTAE v4.03
Wang, N^d^	Journal	Blood	2019	ChiCTR-OPN-16008526	a pilot study	38	CF	CAR19–5.1 ± 2.1*10^6/kg; CAR22–5.3 ± 2.4*10^6/kg	CD19 + CD22	CD28 + 41BB	CTCTAE v4.03
Zhou, X	Journal	Frontiers in Immunology	2020	ChiCTR-OOC-16007779)	phase 1	21	CF	8.9(0.3–48)* 10^5/kg	CD19	forth generation	CTCTAE v4.03
Wang, Jia	Journal	British Journal of Haematology	2020	ChiCTR-ONN-16009862+ ChiCTR1800019622	a pilot study	23	CF	1*10^6/Kg	CD19	41BB	CTCAE v4.03
Zhiling Yan	Journal	Lancet Haematol	2019	ChiCTROIC-17,011,272	phase 2	21	CF	1*10^6/kg	CD19 + BCMA	41BB	CTCAE v4.0
Bao, F.	Journal	Zhonghua xueyexue zazhi	2018		case-series	10	CF	4.27(0.30–6.93)*10^6/kg	CD19	41BB	CTCAE
Jain, T	Journal	Blood Advances	2020	NCT01044069; NCT03070327; commercial CAR T cells	clinical trials; retrospectively	83	CF/Cy/Bendam	Not found	CD19、BCMA	CD28、41BB	CTCAE v5.0
Popat, R	Abstract	Blood	2019	NCT03287804	phase 1	11	CF	(15 or 75 or 225 or 600 or 900)*10^6	BCMA+TACI	CD28 + OX40	Not found
Usmani, S. Z	Abstract	HemaSphere	2020	NCT03548207	phase 1b	29	CF	0.73(0.5–0.9)*10^6/kg	BCMA	41BB	CTCAE v5.0
Mailankody, S	Abstract	HemaSphere	2020	NCT034330011	phase1/2	51	CF	(300 or 450 or 600)*10^6	BCMA	41BB	Not found
Hu, Jianda	Abstract	Blood	2018	NCT03391726	phase 2/3	8	CF	(0.7–6) *10^6/kg.	CD19	41BB	Not found
Amrolia, Persis J.	Abstract	Blood	2018	NCT03287817	phase 1; AUTO3	8	CF	(1 or 3 or 5)*10^6/kg	CD19 + CD22	OX40(CD19); 41BB(CD22)	Not found
Ardeshna, Kirit	Abstract	Blood	2019	NCT03287817	phase1/2; AUTO3	11	CF	(50 or 150) *10^6	CD19 + CD22	OX40(CD19); 41BB(CD22)	Not found
Yan, Lingzhi	Abstract	Blood	2019	NCT03196414	phase 1/2	28	CF	CD19 1.0*10^7/kg; BCMA(2–6.8) × 10^7/kg	CD19 + BCMA	41BB	Not found
Wierda, William G	Abstract	Blood	2018	NCT02614066	phase 1	35	CF	(0.5 or 1 or 2)*10^6/kg	CD19	41BB	Not found
Topp, M. S.	Abstract	Hematological Oncology	2019	NCT02348216	ZUMA-1 updated	21	CF	2*10^6/kg	CD19	CD28	Not found
Jiang, Songfu	Abstract	Blood	2018			16	CF	(0.5 or 1.8 or 1.5)*10^8	BCMA	41BB	Not found
Dourthe, M. E	Abstract	Blood	2019		sponsored-clinical trial	41	CF	(2–5)*10^6/kg (weight ≤ 50 kg); (1–2.5)*10^8 /kg (weight > 50 kg)	CD19	41BB	Not found
Jacobson, Caron	Abstract	Blood	2020	NCT03105336	phase 2	146	CF	2*10^6/kg	CD19	CD28	CTCAE v4.03
WayneAS	Abstract	HemaSphere	2019	NCT02625480	phase1	24	CF	1 or 2*10^6/kg	CD19	41BB	Not found

^a^The two are from the same article. The co-stimulatory molecule of the former dataset is CD28, and that of the latter dataset is 41BB

^b^21 patients were included in this article, but 19 patients were analyzed for evaluating hematological toxicity

^c^ The two are from the same article. Axicabtagene ciloleucel is used in the former dataset and tisagenlecleucel is used in the latter dataset

^d^The two are from the same article. The former data was focusing on the patients with ALL (acute lymphocytic leukemia) and the latter data was focusing on the patients with NHL (Non-Hodgkin Lymphoma)

**Table 2:** Basic characteristics of the included patients

Name	Disease	Sample	Sex (male%)	Age [median(range)]	Prior therapy lines	HSCT%
Abramson, J. S	lymphoma	269	65%	63(54–70)	≥3 lines: 51%	35%
Zhiling Yan	MM	21	48%	58(49.5–61)	mean lines: 6	14%
Ali, S. A	MM	12			median lines: 7	100%
Cohen, A. D	MM	25	68%	58(44–75)	median(range) lines: 7(3–13)	92%
Curran, K. J	ALL	25		13.5(1–22.5)	Not found	20%
Enblad, Gunilla	lymphoma+ALL	15	47%	61(24–71)	mean lines: 1.73	40%
Fry, T. J	B-ALL	21	62%	19(7–30)	Not found	90%
Gardner, R. A	B-ALL	43	44%	12.3(1.3–25.4)	Not found	62%
Geyer, M. B.	CLL	8	100%	58(45–70)	Not found	
Geyer, M. B	CLL + NHL	20	70%	63(43–75)	median(range) lines: 4(1–11)	0
Goto, H	DLBCL	9	56%	61(32–73)	mean lines; 3	44.40%
Fried, S.	ALL+NHL	35	71%	27(3.5–55)	Not found	37%
Lee, D. W	ALL+DLBCL	19	67%	1 to 30	mean lines: 2	38%
Locke, F. L	lymphoma	108	68%	Phase 1: 59 (IQR34–69);Phase 2: 58 (IQR51–64)	median lines: 3	23%
Maude, S. L	ALL	75	57%	11(3–23)	median(range) lines: 3(1–8)	61%
Xu, J	MM	17	65%	55(40–73)	median(range) lines: 5(3–11)	47%
Schuster, S. J	DLBCL	111	65%	56 (22–76)	≥3 lines: 52%	49%
Raje, N	MM	33	64%	60(37–75)	median(range) lines: 7(3–23)	97%
Schuster, S. J	FCL + DLBCL	28	64%	57.5(25–77)	median(range) lines: 4.5 (1–10)	39%
Wang, N^a^	ALL	51	63%	27 (9–62)	Not found	24%
Wang, N^a^	NHL	38	58%	47 (17–71)	Not found	15.80%
Zhao, W. H	MM	57	60%	54 (27–72)	median(range) lines: 3 (1–9)	18%
Wang, M	MM	68	84%	65 (38–79)	≥3lines 81%; median(range) lines: 3 (1–5)	43%
Sang, W	DLBCL	21	62%	55 (23–72)	median(range) lines: 3(1–6)	5%
Wayne AS,	ALL	24	63%	13(3–20)	≥3 lines: 42%	25%
Ghorashian, S	ALL	14	93%	9.24 (1.35–19.28)	median(range) lines: 4(2–7)	71%
Wang, Jia	ALL	23	61%	42(10–67)	median(range) lines: 2(2–3)	22%
Bao, F.	ALL+NHL	10	40%	33.5(25–69)	Not found	
Hu, Jianda	DLBCL	8		52(27–70)	Not found	
Jiang, Songfu	MM	16		55 (39–67)	median(range) lines: 4(2–10)	56%
Wierda, William G	ALL	35	51%	40(18–69)	≥3 lines: 60%	
Yan, Lingzhi	MM	28	82%	57.5 (42–69)	mean(range) lines: 3(2–8)	
Amrolia, Persis J.	ALL	8		7.5(4–16)	Not found	63%
Ardeshna, Kirit	DLBCL	11		49	median lines: 3	27%
Strati, Paolo	lymphoma	31	74%	52(23–76)	>3lines 45%; median(range) lines: 3(1–11)	35%
Yan, Zi-Xun	NHL	10	80%	47(32–59)	≥3lines: 100%	
Ying, Zhitao^b^	NHL	3	67%	<65	mean lines: 9.7	0
Ying, Zhitao^b^	NHL	3	100%	<65	mean lines: 8	0
Topp, M. S.	lymphoma	21	67%	63 (36–73)	≥2lines: 76%	10%
An, F	ALL	47	49%	22(3–72)	<10lines: 59.6%	19.10%
Dourthe, M. E	ALL	41		18.2(1–29.2)	Not found	63%
Mailankody, S	MM	51		61(33–77)	median(range) lines: 6 (3–18)	
Popat, R	MM	11		61 (45–69)	median(range) lines: 5(3–6)	73%
Ramos, C. A	HL	42	67%	35(17–69)	median(range) lines: 7(2–23)	100%
Sesques, P^c^	DLBCL	33	72%	62 (28–75)	≥4 lines: 64%	30%
Sesques, P^c^	DLBCL	28	57%	59 (27–72)	≥4 lines: 79%	29%
Shah, N. N	lymphoma	22	86%	57 (38–72)	Not found	50%
Tong, C	NHL	28	39%		≥3 lines: 79%	
Usmani, S. Z	MM	29			median(range) lines: 5(3–18)	
Wang, Y	ALL	21	52%	13 (3–69)	median(range) lines: 4(1–7)	9.52%
Zhou, X	NHL + DLBCL	21	62%	31 to 77	≥4 lines: 38%	
Ramos, Carlos A	NHL	16		67(17–73)	Not found	31%
Zhang, W. Y	NHL	11		≥18	Not found	9%
Jain, T	NHL + ALL+MM	83	67%	58(19–85)	Not found	37%
Jacobson, Caron	iNHL	146	57%	61(34–79)	median(range) lines: 3(1–10)	

^a^ The two are from the same article. The former data was focusing on the patients with ALL (acute lymphocytic leukemia) and the latter data was focusing on the patients with NHL (Non-Hodgkin Lymphoma)

^b^ The two are from the same article. The co-stimulatory molecule of the former dataset is CD28, and that of the latter dataset is 41BB

^c^ The two are from the same article. Axicabtagene ciloleucel is used in the former dataset and tisagenlecleucel is used in the latter dataset

**Table 3:** Risk of bias

Study	Selection	Ascertainment	Causality	Reporting	Risk of bias
Ying et al				X	Low
Yan et al				X	Low
Sang et al				X	Low
Tong et al				X	Low
Xu et al				X	Low
Zhao et al				X	Low
Shah et al				X	Low
Wang et al	X			X	Moderate
Fried et al				X	Low
An et al	X			X	Moderate
Ramos et al	X			X	Moderate
Raje et al				X	Low
Abramson et al	X			X	Moderate
Wang et al	X			X	Moderate
Cohen et al	X			X	Moderate
Goto et al	X				Low
Schuster et al	X			X	Moderate
Ghorashian et al	X			X	Moderate
Maude et al				X	Low
Strati et al					Low
Locke et al				X	Low
Fry et al					Low
Ali et al				X	Low
Enblad et al					Low
Schuster et al	X			X	Moderate
Gardner et al				X	Low
Curran et al	X				Low
Ramos et al					Low
Zhang et al					Low
Lee et al			X	X	Moderate
Geyer et al					Low
Geyer et al					Low
Sesques et al	X				Low
Wang et al				X	Low
Zhou et al				X	Low
Wang et al	X			X	Moderate
Yan et al	X			X	Moderate
Bao et al		X		X	Moderate
Jain et al			X		Low

Evaluation of methodological quality. Negative points are denoted with “X”. Score of 0–1 suggests low risk of bias, 2–3 moderate, and 4 high

### Hematological toxicity

#### Overall incidence rate

**Fig. 2:**
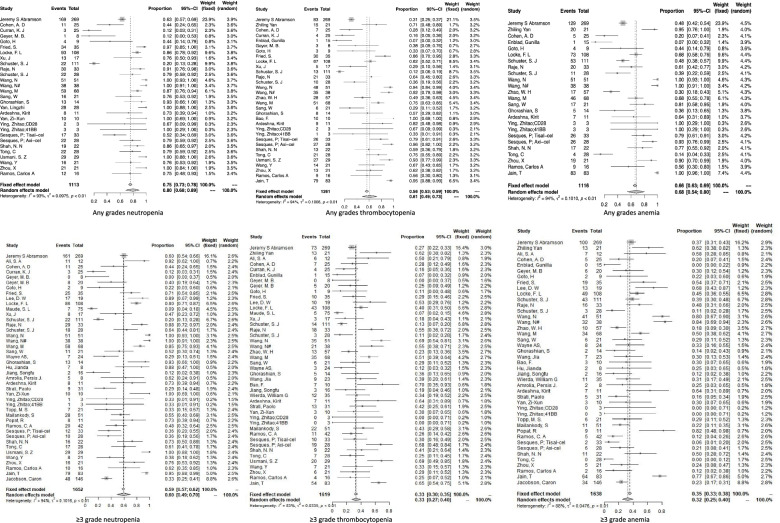
Forest plots of hematological toxicity

Forty-six studies [8, 10–16, 18–25, 27, 28, 30–32, 34, 35, 37–52, 54–56, 58–61] reported the incidence rates of hematological toxicity. Of these, 40 studies [8, 10–12, 14–16, 18–28, 30, 32, 34, 35, 37–42, 44, 46–52, 55, 56, 58, 59] involving 1652 patients explored the rate of neutropenia, 41 [8–16, 18–28, 30–32, 34, 35, 37–46, 48, 49, 52, 54, 56, 59] studies involving 1619 patients explored the rate of thrombocytopenia, and 40 [8–11, 13, 14, 16, 18–25, 27, 28, 30–32, 35, 37, 39–47, 49–52, 54–56, 58, 59] studies involving 1638 patients explored the rate of anemia. As shown in Fig. 2, the total incidences of neutropenia, thrombocytopenia and anemia of any grades were 80% (95% CI: 68–89%), 61% (95% CI: 49–73%), and 68% (95%CI: 54–80%) respectively. And the pooled results of grade ≥ 3 neutropenia, thrombocytopenia and anemia were 60% (95% CI: 49–70%), 33% (95% CI: 27–40%), and 32% (95%CI: 25–40%) respectively. The pooled results are shown in Table 4 in detail.

**Table 4:** overall incidence rate of adverse effects

	Pooled results	95% CI	I^2^
Any grades AEs			
Neutropenia	80%	68–89%	93%
Thrombocytopenia	61%	49–73%	94%
Anemia	68%	54–80%	94%
AST increased	28%	18–43%	92%
ALT increased	30%	26–34%	39%
Serum creatine increased	14%	8–24%	82%
APTT prolonged	56%	31–79%	94%
Fibrinogen decreased	13%	6–22%	72%
Serum creatine increased	14%	8–24%	82%
≥3 grade AEs			
Neutropenia	60%	49–70%	94%
Thrombocytopenia	33%	27–40%	83%
Anemia	32%	25–40%	88%
AST increased	6%	3–10%	51%
ALT increased	2%	1–3%	0%
Serum creatine increased	1%	0–2%	0%
APTT prolonged	4%	1–8%	0%

#### Subgroup analysis

We performed subgroup analysis for age, pathological type, target antigen, co-stimulatory molecule, the proportion of previous HSCT and median lines of prior therapy.

We set the age into three groups as low (< 45 years old), middle (≥45 and < 60 years old) and high (≥60 years old). The pooled results showed younger patients were more likely to experience hematological toxicity but with no statistical significance. According to pathological type, we analyzed the toxicity among patients with lymphoma, leukemia or MM and the result is presented in Tables 5 and 6. Subgroup analysis of target antigen (CD19 vs. no CD19) revealed that non-CD19 cases had the higher rate of hematological toxicity. Especially in analyzing neutropenia, Z test illustrated that the difference between the two groups (CD19 vs. no CD19) was of statistical significance. For neutropenia of any grades, a higher rate of 93% (95% CI: 84–99%) for non-CD19 studies compared with 73% (95% CI: 58–86%) for CD19 studies, and the *P*-value of the Z test was 0.0001. Besides, the analysis of ≥3 grade neutropenia showed that the incidences of non-CD19 cases and CD19 cases was 75% (95% CI: 57–90%) and 52% (95% CI:40–64%) respectively, and the P-value of the Z test was 0.0088. The pooled result of proportion of previous HSCT (< 50% vs. ≥50%) was of no statistical significance. Therefore, the history of HSCT before CAR-T therapy does not have effect on hematological toxicity. Subgroup analysis by prior therapy lines showed that hematological toxicity was less frequent in the case of median lines < 4 compared to ≥4. However, the results were of no statistical significance, except in analysis of any grades thrombocytopenia. Additional details are shown in Tables 5 and 6.

**Table 5:** Subgroup analysis of hematological toxicity

**Any grades of hematological toxicity**								
			Neutropenia		Thrombocytopenia		Anemia	
Median age (years)	< 45	rate^a^	82% (42–100%)	*P* > 0.05	74% (44–95%)	*P* > 0.05	79% (4–100%)	*P* > 0.05
		N^b^	146		156		65	
	≥45 and < 60	rate	82% (64–96%)		57% (39–75%)		77% (59–92%)	
		N	565		605		580	
	> 60	rate	72% (56–85%)		50% (28–71%)		53% (39–68%)	
		N	428		443		443	
Pathological type	leukemia	rate	62% (17–98%)	*P* > 0.05	60% (22–93%)	*P* > 0.05	69% (17–100%)	*P* > 0.05
		N	244		254		176	
	lymphoma	rate	83% (73–90%)		60% (46–73%)		68% (54–80%)	
		N	737		742		721	
	MM	rate	88% (64–100%)		57% (36–77%)		53% (21–84%)	
		N	132		182		136	
Targeting antigen	CD19	rate	73% (58–86%)	*P* = 0.0001	56% (40–71%)	*P* > 0.05	64% (48–79%)	*P* > 0.05
		N	918		933		834	
	non-CD19	rate	93% (84–99%)		70% (54–83%)		74% (46–95%)	
		N	278		328		282	
Proportion of previous HSCT	< 50%	rate	80% (56–97%)	*P* > 0.05	74% (58–87%)	*P* > 0.05	74% (58–87%)	*P* > 0.05
		N	978		1071		973	
	≥50%	rate	77% (62–89%)		52% (34–69%)		49% (23–74%)	
		N	94		94		94	
Median lines of prior therapy	< 4	rate	79% (61–93%)	*P* > 0.05	42% (27–58%)	*P* = 0.0252	55% (43–67%)	*P* > 0.05
		N	673		707		690	
	≥4	rate	81% (69–92%)		67% (53–80%)		65% (43–84%)	
		N	267		288		238	
Co-stimulatory molecule	CD28	rate	88% (82–93%)	*P* > 0.05	79% (59–94%)	*P* = 0.0054	79% (64–92%)	*P* = 0.0274
		N	207		207		207	
	41BB	rate	65% (41–86%)		36% (17–57%)		55% (42–67%)	
		N	463		453		453	
Median age in leukemia cases	< 20	rate	61% (10–100%)	*P* > 0.05	45% (14–79%)	*P* = 0.032	No analysis	
		N	60		60			
	≥20	rate	83% (38–100%)		87% (66–99%)			
		N	94		104			
Median age in lymphoma cases	< 60	rate	85% (63–99%)	*P* > 0.05	59% (35–81%)	*P* > 0.05	80% (64–93%)	*P* = 0.0424
		N	404		394		394	
	≥60	rate	67% (51–81%)		47% (23–72%)		52% (34–69%)	
		N	395		410		410	
**≥3 grade of hematological toxicity**								
			Neutropenia		Thrombocytopenia		Anemia	
Median age (years)	< 45	rate	57% (28–84%)	*P* > 0.05	33% (20–47%)	*P* > 0.05	38% (22–56%)	*P* > 0.05
		N	314		374		261	
	≥45 and < 60	rate	59% (40–76%)		32% (22–43%)		34% (22–46%)	
		N	592		662		645	
	> 60	rate	59% (45–71%)		32% (23–43%)		28% (18–40%)	
		N	531		514		546	
Pathological type	leukemia	rate	48% (22–76%)	*P* > 0.05	28% (16–42%)	*P* > 0.05	41% (28–54%)	*P* > 0.05
		N	390		450		350	
	lymphoma	rate	60% (49–71%)		32% (25–40%)		24% (16–34%)	
		N	985		825		979	
	MM	rate	58% (29–84%)		40% (28–53%)		31% (15–50%)	
		N	215		261		350	
Targeting antigen	CD19	rate	52% (40–64%)	*P* = 0.0088	29% (22–36%)	*P* > 0.05	28% (21–35%)	*P* > 0.05
		N	1313		1221		1267	
	non-CD19	rate	75% (57–90%)		43% (32–54%)		42% (24–62%)	
		N	339		398		371	
Proportion of previous HSCT	< 50%	rate	58% (44–71%)	*P* > 0.05	33% (26–41%)	*P* > 0.05	36% (26–45%)	*P* > 0.05
		N	1093		1180		1146	
	≥50%	rate	59% (34–82%)		30% (16–46%)		34% (19–50%)	
		N	258		239		183	
Median lines of prior therapy	< 4	rate	53% (38–68%)	*P* > 0.05	28% (20–36%)	*P* > 0.05	32% (25–39%)	*P* > 0.05
		N	961		924		999	
	≥4	rate	60% (46–73%)		34% (24–43%)		24% (13–36%)	
		N	419		440		390	
Co-stimulatory molecule	CD28	rate	47% (34–66%)	*P* > 0.05	47% (34–60%)	*P* = 0.0004	29% (18–41%)	*P* > 0.05
		N	405		238		405	
	41BB	rate	53% (38–74%)		18% (10–27%)		22% (11–34%)	
		N	471		463		471	
Median age in leukemia cases	< 20	rate	46% (18–75%)	*P* > 0.05	23% (10–40%)	*P* > 0.05	36% (18–70%)	*P* > 0.05
		N	186		178		65	
	≥20	rate	58% (11–97%)		37% (20–56%)		42% (28–65%)	
		N	114		182		174	
Median age in lymphoma cases	< 60	rate	64% (45–82%)	*P* > 0.05	32% (22–44%)	*P* > 0.05	31% (19–43%)	*P* > 0.05
		N	485		477		485	
	≥60	rate	49% (32–67%)		27% (16–40%)		22% (12–34%)	
		N	562		410		577	
Median age in MM cases	< 60	rate	34% (14–57%)	*P* = 0.0227	29% (16–44%)	*P* = 0.0356	26% (9–48%)	*P* > 0.05
		N	58		136		119	
	≥60	rate	73% (47–93%)		48% (37–58%)		48% (18–79%)	
		N	95		84		95	

^**a**^ Rate means the pooled results and 95% CI of incidence

^b^N means the number of pooled patients in the dataset

**Table 6:** Subgroup analysis of non-hematological toxicity

**Any grades of Coagulation toxicity**						
			APTT prolonged		Fibrinogen decreased	
Pathological type	leukemia	rate	50% (3–97%)	*P* > 0.05	12% (7–41%)	*P* > 0.05
		N	98		118	
	MM	rate	59% (19–94%)		16% (1–41%)	
		N	123		103	
**Any grades of Hepatic toxicity**						
			AST increased		ALT increased	
Pathological type	leukemia	rate	25% (18–32%)	*P* > 0.05	34% (24–44%)	*P* > 0.05
		N	154		93	
	lymphoma	rate	24% (16–34%)		21% (15–27%)	
		N	249		249	
	MM	rate	44% (14–77%)		25% (19–32%)	
		N	120		188	
**≥3 grade of Hepatic toxicity**						
			AST increased		ALT increased	
Pathological type	leukemia	rate	7% (3–12%)	*P* = 0.0016	4% (1–7%)	*P* > 0.05
		N	236		250	
	lymphoma	rate	1% (0–4%)		1% (0–3%)	
		N	249		249	
	MM	rate	16% (9–25%)		1% (0–4%)	
		N	132		200	

For analyzing the effect of age on grade ≥ 3 hematological toxicity in different pathological types, we conducted a subgroup analysis. Considering the distribution of age varying among different cancers, subgroups were set by different age. For studies focusing on lymphoma (< 60 vs. ≥60 years old), the patients with the age < 60 were more likely to suffer hematological toxicity regularly. Especially, the pooled result of any grades anemia was of statistical significance and the *P*-value of the Z test was 0.0424. Given that patients with leukemia were younger than lymphoma and MM overall from our extracted data, we set these patients into two group as < 20 and ≥ 20. The results revealed that the incidences of hematological toxicity were regularly higher in the older cases, and the *P*-value of Z test was 0.032 in any grades thrombocytopenia. For MM, because the studies were not adequate as lymphoma and leukemia, we only performed subgroup analysis by age (< 60 vs. ≥60 years old) for grade ≥ 3 hematological toxicity. The results showed that the hematological toxicity was more frequent in ≥60 cases, and the *P*-values of Z test were statistically significant in grade ≥ 3 neutropenia and thrombocytopenia (0.0227 and 0.0356, respectively). The detailed results are shown in Tables 5 and 6.

Aiming to specifically analyze the effect of co-stimulatory molecule on hematological toxicity, we eliminated the confounding factor targeting antigen and chose the part with the most sufficient data. The selected studies focused on lymphoma patients treated with CAR-T cell targeting CD19, and we explored the different effects of co-stimulatory molecule (CD28 vs. 41BB) with the extracted data. As shown in Tables 5 and 6, the results showed that the hematological toxicity was more frequent in cases where the co-stimulatory molecule was CD28, and the Z tests showed that the differences were significant in analyzing thrombocytopenia and any grades anemia. In other words, the co-stimulatory molecule of CD28 has greater tendency to induce hematological toxic effects than that of 41BB. The conclusion is in line with previous studies reporting that 41BB CAR-T cells resulted in less severe AEs [62].

#### Onset time of hematological toxicity

In this part, we only conducted analysis qualitatively. The study by Fried S et al. [16] reported that the median time to onset of neutropenia was 3 days (range 0–21) and severe neutropenia occurred within a median of 7 days (range 0–63), and they reported that the median time to onset of thrombocytopenia was 0 days (range 0–38) and that of grade ≥ 3 was 5.5 days (range 0–28). That is, hematological occurred early in the process of CAR-T therapy. Besides, Wang J et al. [43] reported that grade ≥ 3 hematological toxicity mostly occurred 5 days after pretreatment. And in general, conditioning chemotherapy was conducted 3–5 days before infusion. It was reported that hematological toxicity after CAR-T was in fact associated with lymphodepleting chemotherapy [25]. However, even though it is pretreatment but not the CAR-T cell itself leading to hematological toxicity in mechanism, since conditioning regimen was an important part of CAR-T therapy procedure, we should conclude that CAR-T therapy was related to the toxicity of blood system. Furthermore, the facts listed above were important reminders for us to note the hematological toxic effects shortly after initiating CAR-T therapy.

#### Recovery time of hematological toxicity

We analyzed hematological toxicity on day 28 and on the 3rd month after infusion. However, because of the limitations of the extracted data, we only explored the grade ≥ 3 cytopenia, neutropenia and thrombocytopenia, and the calculated data is presented in Table 4. On D28 after infusion, the pooled results of grade ≥ 3 cytopenia, neutropenia and thrombocytopenia were 39% (95%CI: 24–55%), 13% (95%CI: 5–25%) and 25% (95%CI: 19–36%) respectively. On the 3rd month, the grade ≥ 3 neutropenia was 5% (95%CI: 0–16%), and grade ≥ 3 thrombocytopenia was 20% (95%CI: 8–35%). Both time points of day 28 and the 3rd month witnessed higher thrombocytopenia than neutropenia. And as shown in Table 4, the overall incidences of neutropenia were more frequent than thrombocytopenia. An explanation is that platelets are more difficult to recover than neutrophils, consistent with the conclusion of one study by Jain T et al. [46]. They demonstrated that hematological count “normalization” (in the normal range for the laboratory) was much easier for neutrophils than hemoglobin and platelets.

#### Sensitivity analysis and publication bias

**Fig. 3:**
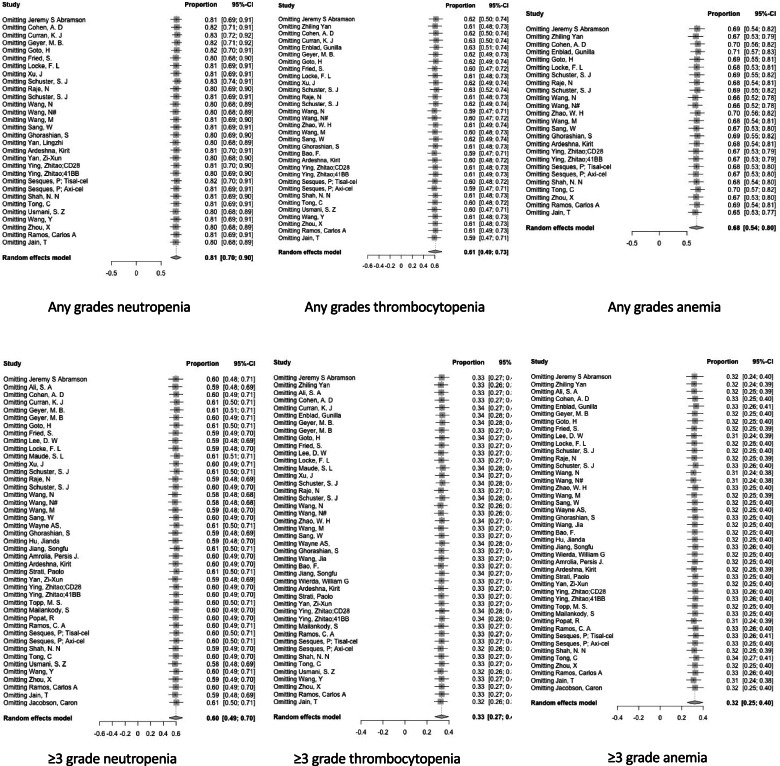
Sensitivity analysis of hematological toxicity

**Fig. 4:**
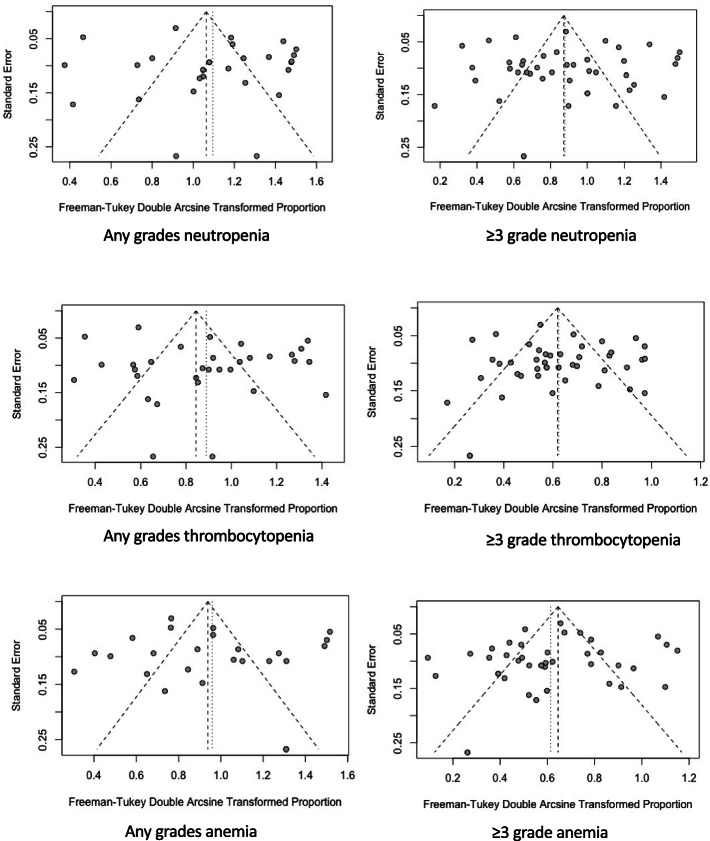
Funnel plots of Egger tests for hematological toxicity

Sensitivity analysis was performed in overall rate of the hematological toxicity. And the results showed that after omitting the studies one by one, the pooled results did not change significantly. In other words, the results of the meta-analysis were stable enough (Fig. 3). Egger test was conducted for analyzing publication bias in evaluating overall incidences of neutropenia, thrombocytopenia and anemia. If *P* value > 0.05 was met in analyzing, it was considered as having no publication bias (data not shown). The funnel plots of Egger tests are shown in Fig. 4. Publication bias did not occur in all six groups.

### Coagulation toxicity

Pooling data of the data indicated that the incidences of any grades APTT prolongation and fibrinogenopenia were 56% (95%CI: 31–79%) and 13% (95%CI: 6–22%) respectively, and that proportion of ≥3 grade APTT prolongation and fibrinogenopenia were 4% (95%CI: 2–79%) and 5% (95%CI: 2–9%) (Table 4). Furthermore, we performed the subgroup analysis of any grades APTT prolongation and fibrinogenopenia by pathological type (just in cases of “leukemia” and “MM”). As shown in Tables 5 and 6, the difference between the two subgroups was not statistically significant. The incidences of APTT prolongation were 50% (95%CI: 3–97%) and 39% (95%CI: 10–73%) in leukemia cases and MM cases respectively. And the pooled results showed that the rates of any grades fibrinogenopenia were comparable in the two subgroups of leukemia (12%) and MM (16%).

### Hepatotoxicity

Meta-analysis showed that rates of any grades AST and ALT increasement were 28% (95%CI: 18–43%) and 29% (95%CI: 24–35%) respectively, and that incidences of grade ≥ 3 AST and ALT increasement were 6% (95%CI: 3–10%) and 2% (95%CI: 1–3%) (Table 4). We also performed subgroup analysis by pathological type in this part and the additional data is presented in Tables 5 and 6 in detail.

### Nephrotoxicity

To explore the effect of CAR-T cell therapy on renal function, we conducted an analysis on data about serum creatine elevated (SCE). As shown in Table 4, the proportion of any grades SCE was 14% (95%CI: 8–24%), and the incidences of grade ≥ 3 SCE were quite low. Given that the extracted data of nephrotoxicity was not rich, we did not perform subgroup analysis in this section.

## Discussion

CAR-T cell therapy has dramatical efficacy in hematological malignancies and is developing continuously. There are many articles exploring the pooled complete remission, and the incidence of CRS, as the characteristic adverse effect of CAR-T therapy. However, no study specifically reported the relevant hematological toxicity, coagulation toxicity, hepatotoxicity and nephrotoxicity. The purpose of our meta-analysis was to fill this gap and the main aim was evaluating hematological toxicity after CAR-T infusion.

This meta-analysis showed that the incidence rate of grade 3/4 neutropenia, thrombocytopenia and anemia were 60, 33 and 32%, respectively during CAR-T treatment. For lymphoma, these incidences were 60, 32 and 24% correspondingly. For leukemia, they were 48, 28 and 41% correspondingly. For MM, they were 58, 40 and 31% correspondingly. Compared with grade 3/4 CRS from previous reviews [63–65], our pooled results indicated that the most common grade ≥ 3 AEs were hematological toxic effects. Based on I^2^ statistic, the results from random-effect model were used to represent overall hematological toxicity. At the same time, subgroup analysis did not reduce heterogeneity. According to subgroup analysis and the corresponding Z test, hematological toxicity is more frequent in younger patients, in patients with ≥4 median lines of prior therapy and in cases targeting CD19. With specific regards to anti-CD19 CAR-T cell constructs, we focused on lymphoma to explore the difference of hematological toxicity between CD28 and 41BB, as two main co-stimulatory molecules in CAR-T therapy. Consistent with our expectations and similar with other AEs, hematological toxicity was more likely to occur in CD28 cases [62]. Some studies reported that patients with severe neutropenia died from severe infections, and some patients with severe thrombocytopenia died because of intracranial hemorrhage or other life-threatening bleeding events [11, 21, 28, 43, 44, 66]. In long-term follow-up after CAR-T therapy, most delayed hematological toxicities were not life-threatening and would ameliorate 3 months after treatment [28, 46]. This reminds us of paying attention to hematological toxicities in the early process of CAR-T therapy. Hepatotoxicity, nephrotoxicity and coagulation disorder are less frequent, compared with hematological toxicity, CRS and ICANS. All of these AEs can reflect the levels of inflammation in patients treated with CAR-T cell, and this meta-analysis provided the pooled results to clinicians for reference.

Cytopenia was common after CAR-T cell infusion. Meanwhile, some studies reported that myelodysplastic syndrome (MDS), characterized as cytopenia, occurred 4–39 months after infusion [27, 28, 46, 67–69]. The mechanism of cytopenia is unclear currently, and it was important to rule out the process of CAR-T therapy or MDS as the cause of cytopenia [68]. However, Strati P et al. reported that cytopenia at day 30 after infusion was not associated with the later diagnosed MDS statistically [27]. The conclusion denied the association between cytopenia and MDS to some extent. Meanwhile, Jain T et al. deemed that inflammation factors remained significantly associated with hematopoietic recovery at 1 month [46]. In other words, the viewpoints about cytopenia were not consistent. Besides, whether MDS is secondary to CAR-T therapy also remains unclear, although some researchers held the standpoints that MDS were attributed to previous chemotherapies [27, 28]. To figure out the potential mechanism of cytopenia or MDS, more work exploring its etiology is needed.

Cytokine release is a double-edged sword as high cytokine levels can result in severe AEs [70]. CRS, the most common toxicity of CAR-T cell therapy, is triggered by engagement of their CARs with the antigen expressed on tumor cells [3]. Hematological toxicities potentially leading to additional complications such as infection or hemorrhage are also associated with cytokine release after CAR-T cell infusion. The study published recently proposed that improved CRS management may improve hematopoietic recovery following CD19 CAR T-cell therapy [4]. Management for CRS and ICANS has been specialized and the related guideline is being constantly being optimized. As hematological toxicities often occur after lymphodepleting chemotherapy, antiviral prophylaxis, i.e. acyclovir, should be started with pretreatment. Antimicrobial and antifungal prophylaxis may be considered when severe or persistent neutropenia happened [71]. Additionally, extended growth factors and transfusional support are needed for hematopoietic recovery [4, 72]. Meanwhile, symptomatic treatment, such as antibiotics and rehydration therapy, and professional nursing are important as well.

CAR-T cell therapy has achieved dramatical efficacy in ALL, B cell lymphoma and MM, but not in acute myeloid leukemia (AML). What limited the use of CAR-T cell in AML is the absence of specific antigen, as many myeloid antigens also expressed on hematopoietic stem cells which would lead to myelosuppression [3, 73]. Therapeutic approach still needs to be optimize to improve the efficacy and safety of CAR-T cell therapy, such as questing more specific antigens, improving CAR structure, professional management during the CAR-T therapy and application of combination of CAR-T cell and other therapies [71, 72, 74]. Recently, the clinical study showed that CD19-directed CAR-T cell with concurrent ibrutinib for relapsed/refractory (R/R) chronic lymphocytic leukemia (CLL) led to high rates of MRD-negative with low CRS severity [75].

Compared with previous meta-analysis about CAR-T treatment, the study holds some advantages. We included more studies and targeted not only a single pathological type. Besides, we aimed to analyze hematological toxicity during CAR-T therapy, which was not reported by other systematical reviews. Thirdly, we performed subgroup analysis by age, pathological type, targeting antigen, co-stimulatory molecule, proportion of HSCT and median lines of prior lines. In addition, we also analyzed hepatotoxicity, nephrotoxicity and coagulation disorder, all of which should be paid attention to but have not been explored previously.

This meta-analysis has some limitations as well. Firstly, we defined all kinds of lymphoma (DLBCL, MCL, HL, etc.) as “lymphoma”, and we set all kinds of leukemia into the “leukemia” subgroup. Some studies pooled all patients with different pathological types together and analyzed the efficacy and safety of CAR-T therapy. When extracting the data in this situation, we deemed the subgroup as the pathological type in majority of the patients included in the study. For example, the study by Shah N. N. et al. [14] included 11 DLBCL patients, 7 MCL patients, 1 FCL patient and 3 CLL patients, so we categorized them as “lymphoma”. This method of classification biased the pooled results. Secondly, some articles provided mean lines but not median lines of prior therapy. According to the statistics principle that both mean and median stand for the central tendency of the relevant data, we deemed the mean lines as the corresponding median lines roughly. Additionally, we included some conference proceedings to extract data for analyzing. The data was not detailed as those published in journals, and it might bring bias.

## Conclusions

In conclusion, the CAR-T therapy is associated with hematological toxic effects. And some cases died from infections or severe hemorrhage in early period. In long-term follow-up, the majority of hematological toxicity is less life-threatening and most patients will ameliorate after 3 months. However, more work is needed to explore its mechanism. The significance of this study is to provide the pooled results to clinicians for reference, and to remind them of paying attention to prevention and intervention for hematological toxicity in the early process of CAR-T therapy.

## Supplementary Information

**Additional file 1.** PRISMA  Checklist.

## Data Availability

All data generated or analyzed during this study are included in this published article.

## Abbreviations

CAR: Chimeric antigen receptor-modified
HSCT: Hematopoietic stem cell transplantation
AST: Aspartate transferase
ALT: Alanine transaminase
APTT: Activated partial thromboplastin time
CI: Confidence interval
MM: Multiple myeloma
scFv: Single-chain variable fragment
ICANS: Immune effector cell-associated neurotoxicity syndrome AE: adverse effect
CRS: Cytokine release syndrome
SCE: Serum creatine elevated
MDS: Myelodysplastic syndrome
DLBCL: Diffuse large B cell lymphoma
MCL: Mantle cell lymphoma
HL: Hodgkin lymphoma
FCL: Follicular cell lymphoma
CLL: Chronic lymphocytic leukemia
R/R: Relapsed/refractory
